# Age-Friendly Community Interventions for Health and Social Outcomes: A Scoping Review

**DOI:** 10.3390/ijerph20032554

**Published:** 2023-01-31

**Authors:** Andy Hong, Jessie Welch-Stockton, Ja Young Kim, Sarah L. Canham, Valerie Greer, Michelle Sorweid

**Affiliations:** 1 Healthy Aging and Resilient Places (HARP) Lab, College of Architecture + Planning, University of Utah, Salt Lake City, UT 84112, USA; 2 Department of City & Metropolitan Planning, College of Architecture + Planning, University of Utah, Salt Lake City, UT 84112, USA; 3 College of Social Work, University of Utah, Salt Lake City, UT 84112, USA; 4 School of Architecture, College of Architecture + Planning, University of Utah, Salt Lake City, UT 84112, USA; 5 Aging Brain Care Program, University of Utah Health, Salt Lake City, UT 84132, USA; 6 Division of Geriatrics, Department of Internal Medicine, University of Utah Health, Salt Lake City, UT 84132, USA

**Keywords:** age-friendly cities and communities, age-friendly intervention, health outcomes, social outcomes

## Abstract

To address the numerous challenges associated with aging, increased attention has been given to the development of age-friendly cities and communities (AFCC) to promote healthy aging and social participation. However, limited evidence exists for addressing both health and social needs through the AFCC framework. We address this gap by conducting a scoping review of the interventions that target both health and social outcomes within the purview of the AFCC framework. The results showed that many of the successful interventions used a partnership model and behavioral change theories to inform the program design and implementation. The results also indicated that social participation and engagement played a key role in making the interventions successful. However, the results revealed that the literature is dominated by person-focused approaches. Future research should focus more on evaluating environmental-focused interventions and develop a holistic framework that combines both person- and environment-based approaches to healthy aging.

## 1. Introduction

Globally, there are about 720 million people aged 65 years or over, and that number is expected to double in the next three decades [1]. This means that one in five people in the world will be classified as an older adult by 2030, which has important implications for population health. The risk of developing chronic health conditions increases as individuals age, placing an increasing burden on health systems [2]. The physical and emotional burden of providing care to an aging population is compounded by the fiscal burden, threatening the sustainability of health care and social welfare programs [3]. The COVID-19 pandemic has further exacerbated the increasing health care burden associated with aging, and has disrupted older people’s daily routines for meeting critical health care needs, such as dialysis [4]. Older people have been challenged by the increased home confinement, the lack of physical and social contact with family members, friends, and colleagues, the temporary cessation of employment and other activities, and the anxiety and fear of illness and death [5].

As people age, many experience a myriad of changes impacting their health and functional ability to manage independent living in their homes and communities [6]. Moreover, age-related changes, such as physiological and cognitive decline, may lead to mobility limitation and disability [7], which are associated with decreased health status and quality of life [8,9]. These challenges involve not only individual circumstances, but also barriers and facilitators in the social and physical environments, often conceptualized as micro-, meso-, and macro-level environmental factors in the socio-ecological model [10]. The conceptualization of healthy aging, therefore, comprises physical needs (e.g., mobility status), cognitive capabilities (e.g., resilience), and environmental adaptations to enhance functioning in the context of the community (e.g., non-slip flooring). The socio-ecological model further underscores the broader role of supports and services to facilitate aging well in the community [10].

In response to the challenges associated with aging, the World Health Organization (WHO) has initiated the global movement for promoting age-friendly cities and communities (AFCC) that provides a mechanism and policy framework to develop a place-based approach to healthy and active aging. Age-friendly communities are important because they not only provide the services and infrastructure to promote health, but also address other serious issues, such as poverty and social isolation. In fact, one of the primary goals of the AFCC framework is encouraging social participation [11]. The social participation aspect has become even more critical during the COVID-19 pandemic as social distancing measures have exacerbated social isolation and loneliness among older adults [12]. However, most of the existing literature focuses on either health outcomes or social outcomes, providing little evidence on interventions that target both. We fill this gap by conducting a scoping review of peer-reviewed studies that examine community-based interventions targeting both health and social outcomes within the purview of the AFCC framework. For a comprehensive review, the paper includes all the relevant community-based interventions, programs, and policies that follow the general principle of the AFCC model. The findings of this review will help inform policymakers in developing and implementing evidence-based, locally relevant policies and strategies to move towards creating an age-friendly environment.

## 2. Materials and Methods

### 2.1. Research Design

For this scoping review, we utilized the framework outlined by Arksey and O’Malley [13] to perform article selection in five stages: (1) identifying the research question; (2) identifying the relevant studies; (3) study selection; (4) charting the data; and (5) collating, summarizing, and reporting the results. For transparency and reproducibility, we adhered to the PRISMA-ScR (Preferred Reporting Items for Systematic Reviews and Meta-Analyses extension for Scoping Reviews) guidelines in reporting the results.

### 2.2. Research Question

We examined the following research question: What are the health and social outcomes of Age-Friendly Cities and Communities (AFCC) interventions for older adults, worldwide? Older adults were broadly defined as people aged 60 years or older, but in some cases we included studies that used 50 as the lower age cut-off to broaden our search. Age-friendly community interventions were defined as policies, environmental approaches, promotions, education, and multi-component strategies (e.g., interventions that combine multiple environmental, educational, programming strategies) in community settings, rather than in institutional settings. The definition of a community is a group of people who share distinctive characteristics associated with a common geography (e.g., living in the same neighborhood), or similar identities or interests [14]. We excluded studies that examined individual interventions involving the direct delivery of personal health care services, medical treatments, or counseling to individuals in institutional settings, such as hospitals, nursing homes, and assisted living facilities. We excluded studies that did not report both health and social outcomes. Our definition of health outcomes included both specific definitions of physical and mental health, often used by medical professionals (e.g., chronic diseases and cognitive disorders), and a broader definition of physical, mental, and social well-being, used by WHO [15], which includes general health status, functional abilities, life satisfaction, and quality of life. The social outcomes were measured by increased social participation, social activity, and reduced loneliness and social isolation [16,17].

### 2.3. Search Strategy

We searched for English language peer-reviewed journal articles published from all countries between January 2001 and October 2021. The databases searched for the current study included Web of Science, Scopus, ProQuest, Cochrane Library, Embase, AgeLine, CINAHL, CINAHL Complete, Medline, and Medline Complete. We used four keyword groups to capture the related terms for the search (Table 1). This keyword grouping enabled us to find the research that incorporates four broad topics: age-friendliness, interventions, older adults, and outcomes. The first group was related to age-friendliness, which included terms such as “age-friendly” and “active aging.” The second group included terms related to ‘intervention’ or ‘policies,’ which were intended to capture studies that involved some form of intervention. The third group included terms related to capturing studies that target older adult populations (e.g., senior, elder, and geriatric). The fourth group included terms related to outcomes as our goal was to capture studies that report on both the health and social outcomes of an intervention, initiative, or policy. Based on these terms, we created a search string using an “AND” Boolean operation and built a search query/code for each database (Table A1 in Appendix A).

### 2.4. Study Selection

**Figure 1:**
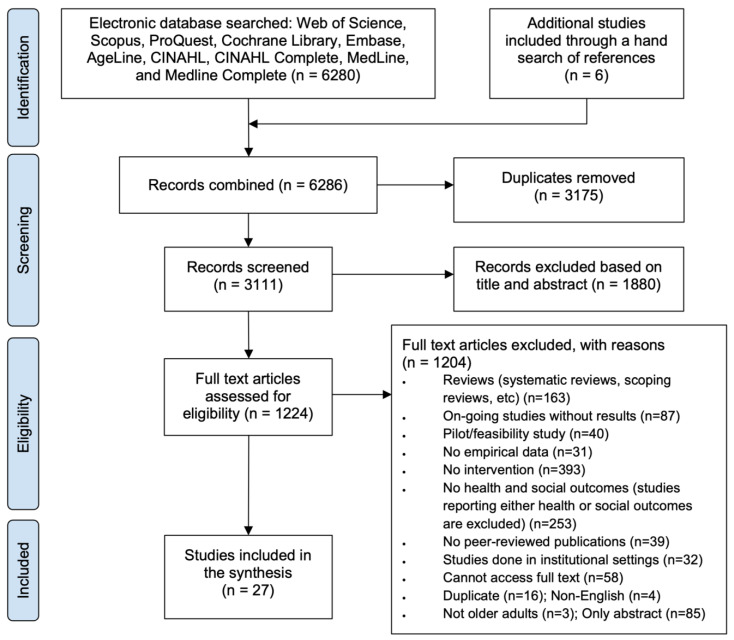
Study selection flow diagram based on PRISMA.

In the first stage of study selection, a total of 6286 records were identified: 6280 records from seven bibliographic databases and six records through a manual search of the references (see Figure 1). For the initial screening, two independent reviewers screened all the records individually, and if there were any conflicts between the reviewers, a third reviewer resolved the conflict.

**Figure 2:**
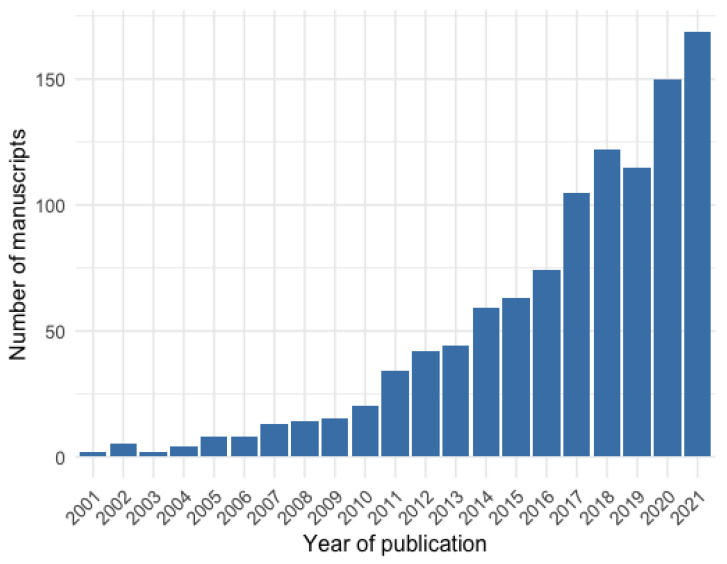
Publication trend from January 2001 to October 2021.

After removing the duplicates (*n* = 3175) and screening based on the title and abstract (*n* = 1880), 1231 records were retained. With the initial screened records, we examined the number of articles by publication year to provide an overall research trend (Figure 2). The trend revealed a gradual increase in the number of publications, with notable increases in publications during three periods (first period: 2010–2011; second period: 2016–2017; third period: 2019–2020).

We then assessed the remaining 1231 records for eligibility through reviewing the full-text articles based on our inclusion and exclusion criteria (Table 2). Although many studies included either health or social outcomes, for the purpose of this review, articles were required to state both health and social outcomes as a result of the implemented intervention. The full-text review was performed using the same procedure as the first screening process which involved two primary reviewers and a third reviewer for resolving conflicts.

Through the full-text review, 27 publications that met the inclusion/exclusion criteria were selected. We extracted the findings from each study and summarized the scope of the existing research on age-friendly community interventions. In the results below, we first describe the characteristics of the studies by synthesizing their findings based on the different intervention types.

## 3. Results

### 3.1. Summary of Selected Studies

A summary of the selected studies used for this review is shown in Table 3. The results were organized by intervention type: physical activity, education, multi-domain, and other. The most common interventions were physical activity (*n* = 10) and educational (*n* = 10), followed by multi-domain (*n* = 5) and other (*n* = 2). Within the ‘other’ intervention type (*n* = 2), one intervention involved transportation, while the other used technology to improve health and social outcomes.

**Figure A1:**
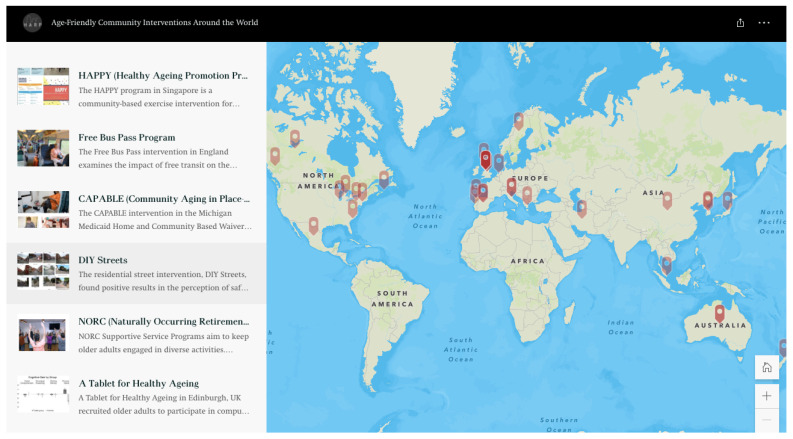
Interactive story map showing some of the key age-friendly community interventions around the world (https://www.harp.utah.edu/storymaps) (accessed on 2 January 2023).

The studies included in this review (*n* = 27) were published from 2008 to 2021. The studies implemented interventions in various countries, with no single country having a substantial amount: United States (*n* = 4), United Kingdom (*n* = 2), Australia (*n* = 3), Canada (*n* = 3), Spain (*n* = 4), Sweden (*n* = 2), Greece (*n* = 2), Singapore (*n* = 1), China (*n* = 1), Portugal (*n* = 1), Mexico (*n* = 1), South Korea (*n* = 2), New Zealand (*n* = 1), Iran (*n* = 1), Japan (*n* = 2), Italy (*n* = 2), Germany/Austria (*n* = 1). To improve knowledge sharing with the general public, we created an interactive story map showing some of the key age-friendly community interventions identified from the scoping review (Figure A1 in Appendix A).

The sample sizes of the studies ranged from 10 to 18,453 participants. While most studies had a majority female sample (*n* = 23), there were multiple studies that did not report the demographic information related to gender (*n* = 4). Female participants represented 52.0% of the sample, meanwhile males only represented 38.6% of the sample, and 9.4% was unknown or unreported.

### 3.2. Physical Activity Interventions

The health and social outcomes linked to physical activity interventions varied in both intervention type and final outcomes (Table 3). The physical activity interventions included in our study were categorized as physical activity (*n* = 3) [19,22,26], exercise programs (*n* = 4) [21,23,24,25], resistance training (*n* = 1) [18], dancing (*n* = 1) [27], and Tai Chi (*n* = 1) [20]. Only one of these [24] had a peer-led intervention, while the remainder were led by trained researchers, professionals, or volunteers. The primary outcomes of the interventions included an increase in physical activity [22,23,26], physical health/functioning [18,20,21,24,25,27], and social functioning [20,21,23,26,27], as well as decreases in social isolation [21,24] and mental health symptoms [24,25,27].

We only found three studies [18,20,27] that examined specific types of exercise in relation to health and social promotion for older adults as opposed to broader fitness or physical activity programs. Only two of these studies [20,27] had significant health and social outcomes. Liao et al. [20] used a Tai Chi intervention with a musical component to create a mind–body practice amongst older adults. This intervention had significant overall increases in physical, psychological, social, and environmental domains (WHOQOL-BREF). Douka et al. [27] implemented a Greek traditional dance intervention and found significant improvements in attention (S4viac/S4viti), verbal fluency (Verflx, Verfls, Verfmo), executive functioning (FUCAS), anxiety, physical condition (arm curl, chair stand, back scratch, 2 min step, timed up-and-go, sit and reach, balance on one leg), and quality of life (WHOQOL). The qualitative research included in the category of physical interventions (Table A3 in Appendix A) stressed the importance of fostering social connectivity in physical activity programs [26]. Without successful socialization, it appeared that many participants reported no change in their feelings of depression and isolation [26].

### 3.3. Educational Interventions

The educational interventions studied in our review varied in both health and social outcomes (Table 3). The articles also differed in terms of the type of education offered and the duration of the intervention. Amongst the educational interventions, six [28,29,31,35,36,37] of the reviewed articles had professional-led health promotion programs where either researchers or trained educators delivered the materials. The most common benefits of these interventions included an increase in physical activity [31,36] physical health [28,35], socialization [28,35,36,37], and cognitive functioning [28,29,32,37], and decreased loneliness [36]. The peer-led programs [30,33] also saw positive results, including improvements in health-related quality of life and decreased loneliness [33], as well as increased self-perception and overall brain health [30]. Other educational interventions included a university education program [29], a family-based empowerment program [34], and a comedy improvisation course [32]. Improved mental health [32], physical health [29,34], cognitive functioning [29,32,37], and social functioning [29,32,34] were commonly cited benefits in these studies.

Our review included both quantitative and qualitative studies. While all the quantitative studies (Table A2 in Appendix A) reported both health and social outcomes, five studies [28,29,30,31,34] reported statistically significant results. Out of those studies, the majority reported on health promotion programs [28,31]. Despite having different curricula and study timeframes, the health promotion programs showed promise in their outcomes. Dumitrache et al. [28] found a significant increase in cognitive performance (MEC/MMSE, CDT) and psychological health (WHOQOL-BREF, psychological), and improved environment (WHOQOL-BREF, environment) amongst older adults who attended a health promotion program. These findings are supported by qualitative research conducted by Sims-Gould et al. [36], where participants reported physical activity as “enjoyable” and “beneficial,” and reported a decrease in social isolation and loneliness after the intervention (Table A3 in Appendix A).

### 3.4. Multi-Domain Interventions

Studies that examined the implementation of interventions that combine multiple program domains, such as physical activity, educational, and group-based programs, were characterized as multi-domain interventions (Table 3). A total of five studies were included in this category—two were qualitative and three were quantitative. The interventions ranged from aging-in-place service programs [38,39,40] to multi-center health promotion programs [41,42]. The common outcomes of these multi-domain interventions were increased physical health [40,41] and social functioning [38,39,40]. Out of the five studies in this category, only two [38,41] reported statistically significant health and social outcomes. Castle et al. [38] implemented a service program for older adults living independently to improve access to preventative and healthy aging services. The researchers found a significant increase in physical activity (one or more times weekly), happiness, and engagement in preventative care, as well as a decrease in muscle weakness (according to a wellness health screen). The qualitative data reported by Greenfield et al. [39] suggests that older adults viewed supportive services within naturally occurring retirement communities (NORC) as vital when aging-in-place, and that service utilization contributes to social determinants, leading to higher participation levels (Table A3 in Appendix A).

### 3.5. Other Interventions

Other interventions included in our study were two studies that did not fit within the categories identified above (Table 3). First, Ballesteros et al. [43] implemented an information and communication technology (ICT) intervention to assist older adults with healthy aging at home. This ICT intervention included both technology training and web-based social networking. Ballesteros et al. [43] found that the technology-based intervention produced significant health and social outcomes, including improved cognitive function (MMSE), behavioral confirmation (SPF-IL scale), and perception of social status (SPF-IL scale). The other study, by Reinhard et al. [44], implemented free bus passes for community-dwelling older adults and found significantly decreased depressive symptoms and loneliness, increased volunteering, and regular contact with friends and children [44].

## 4. Discussion

Over the past several decades, cities and communities have adopted the AFCC framework and implemented a range of interventions to promote healthy aging. While the AFCC framework provides a general direction for interventions, its implementation and evaluation rely heavily on the capacity and the context of each municipality that adopts this framework, resulting in limited empirical evidence on the effectiveness of the age-friendly community interventions. In this scoping review, we identified 27 peer-reviewed journal articles that examined the effect of various age-friendly community interventions on both health and social outcomes in community-dwelling older adults. As noted in previous reviews [45,46], there was a challenge in defining the age-friendly community interventions, implying a possible disconnect between research and practice. To be more comprehensive, we expanded our search to find all relevant age-friendly community interventions, not just the interventions of the WHO’s AFCC programs. Therefore, most of the articles included in our study did not explicitly adopt the WHO’s AFCC framework but addressed similar issues, such as community participation and engagement. Despite these challenges, the results revealed some common themes about the different interventions and their effectiveness on the health and social outcomes of older adults. This review consistently found that the age-friendly community interventions broadly contributed to substantial health outcomes, showing promising results with regards to older adults’ functional and cognitive abilities. This review also identified gaps in the empirical evidence needed to enhance the health and social outcomes of older adults through age-friendly community interventions.

While the AFCC framework tends to emphasize population-based, ecological approaches to healthy aging, our results revealed that age-friendly interventions targeting health and social outcomes seem to be dominated by person-focused approaches, such as physical activity and educational interventions. During the initial search, many studies examining environmental interventions were found; however, most of this research did not meet our inclusion/exclusion criteria, such as reporting both health and social outcomes. On the one hand, this shows that person-focused interventions are strongly represented in the age-friendly interventions targeting health and social outcomes. On the other hand, it is possible that this result could be driven by our eligibility criteria, which required studies to evaluate both the health and social outcomes of age-friendly community interventions. For example, several studies from the CAPABLE project [47,48] found significant results of home modifications in terms of reducing falls and fall-related injuries among older adults. However, this research did not explicitly report any social outcomes, such as social isolation or loneliness. We argue that this is a missed opportunity because housing interventions could be combined with psychosocial interventions that seek to address loneliness and depression among older adults. Previous research has shown that home visiting and counseling services can help build social support and friendship to combat isolation and loneliness among older adults [49]. This indicates a need for developing environmental interventions (e.g., home modification) that address both health and social outcomes through a participatory design approach, engaging communities and stakeholders throughout all stages of the process [50].

Our results suggest that many of the successful age-friendly community interventions [19,22,25,26,33] used a partnership model where a project team formed a strong partnership with the stakeholders and delivery partners, leading to increased uptake of and adherence to the interventions. McKay et al.’s [22] community partnership model proved an effective mode of delivery as more than 500 participants engaged in the physical activity intervention in 26 different locations in British Columbia, Canada. Their project team took a community partnership approach and acted as the ‘experts’ leading training modules and providing consultation to the stakeholders [22]. The stakeholders then delivered 56 programs over the span of one year and five months and saw improvements in both health and social measures [22]. Due to the stakeholder participation and the number of locations available to participants, the intervention was able to cast a wide net and serve older adults who otherwise may have been unable to participate [22]. This suggests that participatory methods and co-design approaches may be key to sustaining and maximizing the impact of interventions [51,52]. To build a strong partnership that can lead to a sustained impact, it may be necessary to move beyond traditional health intervention models and emphasize the benefits beyond health impacts (e.g., social and economic benefits). Interventions can also benefit from adopting existing translational frameworks. For example, RE-AIM is a widely adopted research translation framework that uses a staged approach to evaluating a program’s success through five dimensions: reach, effectiveness, adoption, implementation, and maintenance [53]. Researchers and practitioners should consider using these emerging translational frameworks to effectively monitor and evaluate implementation impacts over time while assessing the evidence of implementation success.

Another notable finding of this review was that several effective age-friendly community interventions used behavioral change theories and frameworks to inform the program design and implementation [19,33,34]. Understanding the underlying mechanisms of interventions is important for identifying the levers of change and best practices for sustained behavioral change. Interventions designed and developed based on well-theorized frameworks could be more effective and easier to adapt than interventions with little or no theoretical basis [54]. In our review, some studies described health behavior models more explicitly than others, and incorporated specific behavior change models into developing their interventions. The physical activity programs and educational interventions reviewed in this study often integrated specific behavioral models to inform the program implementation compared to the other types of interventions. For example, Haynes et al. [19] used the COM-B model which focuses on capability, opportunity, and motivation to influence physical activity behavior. Rabiei et al. [34] relied on Bandura’s theory of self efficacy to focus on the empowerment of older adults when developing a family-based educational intervention. The peer education program in New Zealand [33] built on behavioral change theories, such as the theory of reasoned action, diffusion of innovation, and social learning theory. These theories were applied when developing the core program components that value indigenous knowledge and culture. With multi-domain and other types of interventions, there was little evidence that the appropriate theoretical models were integrated into the program design and implementation. Therefore, a clear challenge for future age-friendly community interventions would be to develop an integrated framework that brings together different theoretical frameworks and models to strengthen and complement the design, implementation, and evaluation of various types of intervention at individual, community, and system levels.

The importance of social connectivity is thoroughly explained in previous research as it can serve as a basis for creating an age-friendly community [55,56]. Consistent with previous research, we found that social participation and engagement played a key role in making age-friendly community interventions successful or unsuccessful. In the case of a telehealth physical activity program implemented in two low-income older adult communities, participation in the intervention varied significantly according to the participants’ social capital [26]. One site maintained a good sense of community, which led to higher uptake of the interventions. The other site, however, had little socialization, negatively impacting their participation in the program. Similarly, in another study that examined older adults’ participation in the NORC programs in New York, USA, the older adults’ relationship with the program as a whole influenced their involvement [39]. The authors noted that focusing solely on service provision or the beneficial aspects of the services may not lead to meaningful involvement. The findings of these studies suggest the importance of developing careful strategies to establish meaningful engagement with older adults and that age-friendly community interventions should incorporate socialization as a basic element of the program design and implementation.

An ongoing challenge with age-friendly community interventions is how to promote social participation and engagement with older adults. This review provided many insights in this regard. First, as noted in Greenfield and Fedor [39], it is critical to understand the dynamic nature of older adults’ needs, relationships, and perceptions of the benefits of interventions in order to ensure high levels of involvement. The benefits may not only be useful in monetary terms but also in other less quantifiable dimensions, such as trust, reciprocity, responsibility, and reputation [57,58]. Second, there needs to be more emphasis on encouraging older adults’ involvement in the program design and delivery, potentially through volunteering and peer-led approaches. The studies included in our review [22,24] demonstrated that interventions that engaged older adults through co-design and co-creation approaches were more likely to have sustained impacts. Third, as shown in some of the successful interventions using Tai Chi and Greek traditional dance classes [20,25,27], enjoyment is a key success factor for achieving the higher participation and engagement of older adults. These findings are consistent with previous research that provided empirical evidence that enjoyment can increase activity participation [59]. Although previous research has identified the meaning of enjoyment in physical activity participation [60], the role of enjoyment with other types of interventions has not been fully explored. Future studies need to examine the meaning of enjoyment among older adults to better understand how it can be achieved. Additionally, as reflected in the update to the Livability Index developed by the American Association of Retired Persons (AARP) in 2022, the emerging models of housing need to be considered to reflect the strategies that communities may take to promote healthy aging and social connection. Lastly, developing an effective engagement strategy requires an understanding of the person-environment fit and the heterogeneity of older adults in terms of their needs, preferences, and values [61]. While some older adults work, others travel, and others become dependent. To achieve higher social connections among older adults across age-friendly interventions, more research is needed to identify how different interventions are appropriate at the different stages of aging and for the diverse sub-populations of older adults, especially those who are socially and economically disadvantaged. The findings from this research can help guide the development of tailored strategies to achieve higher levels of social participation and engagement with older adults.

### Limitations

This study has several limitations. First, we included studies that reported both health and social outcomes. The inclusion of social outcomes was necessary to differentiate age-friendly community interventions from other healthy aging interventions and to assess how certain interventions contribute to social connectivity, which is considered as a basic element for creating age-friendly communities [56]. However, this might have excluded studies that focus on either health outcomes or social outcomes. Second, the selection of outcome measurement instruments varied from one study to the next, making it difficult to compare effectiveness across the interventions. Although some studies adopted commonly used instruments (e.g., MMSE, WHOQOL), most relied on their own surveys to measure the outcomes. Lastly, we intentionally searched for literature that used the term “age-friendliness” to capture more recent studies that adopted the WHO’s AFCC framework. Therefore, some older studies that did not explicitly use this term could have been left out of the search.

## 5. Conclusions

Through this scoping review, we discovered common themes surrounding the effect of age-friendly community interventions that target health and social outcomes. We identified 27 peer-reviewed studies published in countries around the world, with methodologies ranging from quantitative to qualitative and mixed methods.

One of the most consistent findings of this review was that age-friendly community interventions contributed to positive health outcomes, especially with regards to older adults’ functional abilities and cognitive functioning. The results indicated that a partnership model and behavioral change theories provide a useful framework for designing and implementing physical activity and educational interventions. Social participation and engagement also played a key role in making the interventions successful and sustaining the impacts of the interventions.

However, the review found that person-focused programs, such as physical activity and educational training, were the most popular types of interventions, suggesting a lack of diversity in the type of programs offered and the methodologies adopted. Further efforts should be directed to monitor and evaluate the effectiveness of environment-focused interventions in order to advance AFCC initiatives. As such, this review provides important insights that may inform policies and practices to promote healthy aging and age-friendly communities.

## Figures and Tables

**Table 1:** Search term development.

Group 1	Group 2	Group 3	Group 4
age-friendly age-friendliness elder friendly healthy aging aging-in-place urban aging active aging healthy ageing ageing-in-place urban ageing active ageing	intervention * initiative * policies policy framework *	older adult senior * aged elder * geriatric *	outcome * evaluation * impact * effect * benefit * evidence *

Note: The asterisk (*) is a search wildcard, representing any group of characters.

**Table 2:** Study inclusion and exclusion criteria.

	Inclusion Criteria	Exclusion Criteria
Study focus	Studies that examine interventions, policies, and initiatives	Studies that do not examine interventions
Study context	Studies conducted in community settings	Studies conducted in institutional settings
Outcomes of interest	Both health and social outcomes	Studies that do not include any health or social outcomes; also studies that report either health or social outcomes only
Types of study	Peer-reviewed journal publications; empirical studies that employed quantitative/qualitative data	Systematic reviews, scoping reviews, books, book chapters, book reviews, commentaries, opinions, abstracts, conference proceedings, dissertation/theses, study protocols, pilot/feasibility studies
Geographic scope	All countries	
Temporal scope	Publications from January 2001 to October 2021	Publications prior to January 2001
Study population	People aged 60 and above	

**Table 3:** Summary of the selected studies.

Authors (Year)	Country	Population (N)	Study Design	Intervention	Health Outcomes	Social Outcomes	Main Findings
***Physical Activity Interventions***							
Ericson et al. (2018) [18]	Sweden	People aged 65+ (N = 32) w: 32, m: 0	Randomized control trial	Resistance training program [P]	Coherence, health-related quality of life	Hope, negative affect	Resistance training amongst female older adults improved hope and contributed to a decrease in negative affects. This intervention appeared to be positively linked with psychological benefits.
Haynes et al. (2021) [19]	Australia	People aged 60+ (N = 32) w: 22, m: 10	Qualitative	Physical activity and fall-prevention program [P]	Physical activity, motivation, goal-setting, fitness, strength, weight loss, fall prevention	Sense of security, companionship, mutual support, encouragement, community	The healthy aging program reported increased physical activity levels and positivity related to physical activity. Social connections encouraged motivation but were not a primary outcome.
Liao et al. (2019) [20]	China	People aged 60+ (N = 112) w: 69, m: 43	Randomized control trial	Tai Chi program with background music [P]	Physical/psychological domains	Social/environmental domains	Tai Chi with music appeared to improve the quality of life for older adults. Physical, social, psychological, and environmental factors also experienced an increased improvement after the intervention.
Martins et al. (2020) [21]	Portugal	People aged 65+ (N = 34) w: 26, m: 8	Non-randomized cluster trial	Exercise program [P]	Functional abilities	Participation, self-efficacy	The strength and balance exercise program improved physical/functional abilities and self-efficacy for exercise. The intervention also serves to improve social participation and reduce the risk of falls.
McKay et al. (2018) [22]	Canada	People aged 60+ (N = 534) w: 411, m: 123	Type 2 hybrid effectiveness implementation	Physical activity program [P]	Physical activity, mobility limitations, sitting time	Social exclusion, loneliness, happiness	The physical activity program contributed to increased mobility, strength, and physical activity. An additional effect of this intervention was a decrease in social exclusion.
McNamara et al. (2016) [23]	Australia	People aged 55+ (N = 32) w: 24, m: 8	Mixed-methods study	Healthy aging activity program [P]	Leisure activities	Social engagement	The healthy aging activity program reported increased social functioning, mental well-being, household/leisure activities, and overall enjoyment and confidence when participating in the intervention.
Merchant et al. (2021) [24]	Singapore	People aged 60+ (N = 569) n/a	Non-randomized cluster trial	Peer-led, dual-task exercise program [P]	Depressive symptoms, frailty, falls	Isolation	The Healthy Aging Promotion Program, a peer and professionally led intervention, promoted increased cognition, physical functioning, balance, perception of health, and a decrease in falls and social isolation.
Noh et al. (2020) [25]	South Korea	People aged 50+ (N = 40) only women	Randomized control trial	Healthy aging program workshop [P]	Physical/mental health	Social behaviors	The SaBang-DolGi walking exercise program improved mental/physical well-being and vitality amongst menopausal women.
VanRavenstein et al. (2018) [26]	United States	People aged 55+ (N = 21) w: 19, m: 2	Qualitative	Physical activity program [P]	Depressive symptoms, exercise	Social participation, community	The telehealth exercise program delivered to two separate community groups produced differing results. One group improved in social connectedness and physical activity, and the other described isolation and depression to be distressing.
Douka et al. (2019) [27]	Greece	People aged 60+ (N = 60) w: 45, m: 15	Program evaluation (pre/post)	Greek traditional dance program [E]	Attention, verbal frequency, executive function, anxiety, physical condition	Quality of life	The Greek traditional dance program improved the cognitive and physical condition as well as the quality of life for both healthy older adults and older adults with mild cognitive impairment.
*Educational Interventions*							
Dumitrache et al. (2017) [28]	Spain	People aged 65+ (N = 86) w: 61, m: 25	Cross-sectional	Cognitive stimulation, crafts, and exercise workshops [E]	Cognition, overall health	Quality of life, social connectedness, environment	The health promotion program, which was comprised of exercise workshops, crafts, and cognitive stimulation, improved cognition, and the perception of psychological health and leisure activity opportunities.
Fernández-Ballesteros et al. (2013) [29]	Spain	People aged 55+ (N = 95) w: 42, m: 53	Evaluation study	University program [E]	Self-perception of aging, wellbeing	Social relationships, activity, negative and positive affects	The university program intervention reported maintaining activity, overall health, cognitive functioning, and an increase in positive affects amongst older adults.
Gough et al. (2017) [30]	Canada	People aged 50+ (N = 51) w: 39, m: 12	Observational study	Peer-led education series [E]	Brain health, information application, awareness of interventions, health-related goal setting	Self-perception	The peer-led health education program led to an increase in engagement with lifestyle and health interventions amongst older adults. Additionally, this intervention improved attitudes towards aging and goal-setting behaviors.
Mendoza-Nunez et al. (2018) [31]	Mexico	People aged 60+ (N = 64) w: 52, m: 12	Pre/post	Healthy aging program workshop [E]	Healthy lifestyle	Self-perception	The healthy aging programming added to improved physiological health in older adults, and self-perception remained constant.
Morse et al. (2018) [32]	United States	People aged 55+ (N = 10) w: 9, m: 1	Qualitative	Comedy improvisation course [E]	Depressive symptoms, mental flexibility, cognitive acuity	Self-development, social skills, communication skills, connection, loneliness	The benefits of the improvisation course included an increased sense of comfort, positivity, self-development/awareness, feelings of acceptance, and improved problem-solving capabilities.
Oetzel et al. (2020) [33]	New Zealand	Average age of participants 70 (N = 180) w: 122, m: 58	Pre/post	Peer education program [E]	Health, health-related quality of life	Support, tribal identity, loneliness	The peer-led education program led to improved social connectedness, connection with supportive services, and a sense of identity within the participant’s tribe.
Rabiei et al. (2013) [34]	Iran	People aged 55+ (N = 64) w: 33, m: 31	Semi-experimental study	Family-based empowerment program [E]	Physical/mental health	Roles, social functioning, quality of life	The family-based empowerment program was found to increase quality of life amongst older adults and self-esteem, while self-efficacy remained constant.
Shinkai et al. (2016) [35]	Japan	People aged 70+ (N = 686) n/a	Program evaluation (longitudinal)	Health education program [E]	Life-space mobility, instrumental ADL, intellectual activity	Social role	The multicomponent health education and integration program contributed to improved functional health and extended life expectancy.
Sims-Gould et al. (2020) [36]	Canada	People aged 65+ (N = 26) w: 18, m: 9	Qualitative	Health promotion program [E]	Physical activity	Social connectedness, isolation/loneliness	The health promotion program encouraged physical activity and social connections, while addressing loneliness and isolation.
Tagliabue et al. (2018) [37]	Italy	People aged 60+ (N = 84) w: 55, m: 29	Non-randomized cluster trial	Multi-domain cognitive training [E]	Cognitive functioning, memory, executive function, attention	Mood, socialization	The multi-domain cognitive training program reported an improvement in older adults in cognitive functioning and non-verbal reasoning. In addition, improved everyday life, mood, and socialization were also cited as benefits of the program.
***Multi-domain Interventions***							
Castle et al. (2008) [38]	United States	People aged 60+ (N = 823) n/a	Program evaluation (longitudinal)	Senior living enhancement program (SLEP) [M]	Physical activity, body function, habits, care, depressive symptoms	Social activities outside the home	The Senior Living Enhancement Program saw improvements in a majority of the “10 Keys to Healthy Aging” including physical activity, general health, and social connectivity.
Greenfield and Fedor (2015) [39]	United States	People aged 60+ (N = 35) w: 25, m: 10	Natural experiment	Naturally occurring retirement community (NORC) supportive service programs [M]	Overall health, well-being	Social connectedness, volunteerism	The NORC supportive service programs found that positive results in health and social outcomes were indicated by the older adult’s relationship with the program, rather than solely their utilization of the program.
Orellana et al. (2020) [40]	United Kingdom	People aged 60+ (N = 23) w: 18, m: 5	Qualitative	Day centers for older adults [M]	Mental well-being/health, physical well-being/health, hygiene	Social participation/involvement, feelings of dignity/control, quality of life, feelings of security	Day centers for community-dwelling older adults had a positive impact on social participation and meaningful occupation.
Park et al. (2011) [41]	South Korea	People aged 65+ (N = 45) w: 27, m: 18	Randomized control trial	Health education and exercise program [M]	Blood pressure, physical activity, health-related quality of life	Self-care, self-efficacy, quality of life (social)	The health education and exercise program improved systolic blood pressure, self-efficacy, mental/physical health, vitality, and social functioning in older adults with hypertension.
Rainero et al. (2021) [42]	Italy, Australia, Spain, Germany, Austria, Japan	People aged 60+ (N = 201) w: 148, m: 53	Randomized control study	Multi-center health promotion program [M]	Depressive symptoms, nutrition, physical/cognitive function, sleep	Social function, quality of life	The multi-domain ICT healthy aging intervention allowed participants in the intervention to maintain their quality of life with no decrease. In addition, participants had improved mood and nutritional habits.
***Other Interventions***							
Ballesteros et al. (2012) [43]	Spain, Sweden, Greece	People aged 65+ (N = 29) n/a	Randomized control trial	Online social network platform with technology training [O]	Cognition, depressive symptoms, mobility and performance	Affection, behavioral confirmation, social status, comfort, stimulation	The ICT intervention had a positive impact on cognitive functioning, feelings of achievement, being treated with positive regard, independence, and self-realization.
Reinhard et al. (2018) [44]	United Kingdom	People aged 60+ (N = 18,453) w: 9564, m: 8124	Natural experiment	Free bus pass [O]	Public transport use, depressive symptoms	Loneliness, volunteering, social contacts	The free bus pass contributed to reductions in depressive symptoms and feelings of loneliness among older people. It also promoted more regular contact with friends and children.

Note: [P] denotes physical activity interventions; [E] denotes educational interventions; [M] denotes multi-domain interventions; and [O] denotes other interventions.

## Data Availability

Not applicable.

## Appendix A

**Table A1:** Search strings used in different bibliographic databases.

Database	Search String
Web of Science	((TI = “age friendly” OR TI = “age friendliness” OR TI = “elder friendly” OR TI = “healthy aging” OR TI = “aging-in-place” OR TI = “urban aging” OR TI = “active aging” OR TI = “healthy ageing” OR TI = “ageing-in-place” OR TI = “urban ageing” OR TI = “active ageing”) AND (TI = intervention * OR TI = initiative * OR TI = policies OR TI = policy OR TI = framework *) AND (TI = “older adult” OR TI = senior * OR TI = aged OR TI = elder * OR TI = geriatric *) AND (TI = outcome * OR TI = evaluation * OR TI = impact * OR TI = effect * OR TI = benefit * OR TI = evidence *)) OR ((AB = “age friendly” OR AB = “age friendliness” OR AB = “elder friendly” OR AB = “healthy aging” OR AB = “aging-in-place” OR AB = “urban aging” OR AB = “active aging” OR AB = “healthy ageing” OR AB = “ageing-in-place” OR AB = “urban ageing” OR AB = “active ageing”) AND (AB = intervention * OR AB = initiative * OR AB = policies OR AB = policy OR AB = framework *) AND (AB = “older adult” OR AB = senior * OR AB = aged OR AB = elder * OR AB = geriatric *) AND (AB = outcome * OR AB = evaluation * OR AB = impact * OR AB = effect * OR AB = benefit * OR AB = evidence *)) OR ((AK = “age friendly” OR AK = “age friendliness” OR AK = “elder friendly” OR AK = “healthy aging” OR AK = “aging-in-place” OR AK = “urban aging” OR AK = “active aging” OR AK = “healthy ageing” OR AK = “ageing-in-place” OR AK = “urban ageing” OR AK = “active ageing”) AND (AK = intervention * OR AK = initiative * OR AK = policies OR AK = policy OR AK = framework *) AND (AK = “older adult” OR AK = senior * OR AK = aged OR AK = elder * OR AK = geriatric *) AND (AK = outcome * OR AK = evaluation * OR AK = impact * OR AK = effect * OR AK = benefit * OR AK = evidence *))
PubMed	((“age friendly”[Title/Abstract] OR “age friendliness”[Title/Abstract] OR “elder friendly”[Title/Abstract] OR “healthy aging”[Title/Abstract] OR “aging-in-place”[Title/Abstract] OR “urban aging”[Title/Abstract] OR “active aging”[Title/Abstract] OR “healthy ageing”[Title/Abstract] OR “ageing-in-place”[Title/Abstract] OR “urban ageing”[Title/Abstract] OR “active ageing”[Title/Abstract]) AND (“intervention *”[Title/Abstract] OR “initiative *”[Title/Abstract] OR “policies”[Title/Abstract] OR “policy”[Title/Abstract] OR “framework *”[Title/Abstract]) AND (“older adult”[Title/Abstract] OR “senior *”[Title/Abstract] OR “aged”[Title/Abstract] OR “elder *”[Title/Abstract] OR “geriatric *”[Title/Abstract]) AND (“outcome *”[Title/Abstract] OR “evaluation *”[Title/Abstract] OR “impact *”[Title/Abstract] OR “effect *”[Title/Abstract] OR “benefit *”[Title/Abstract] OR “evidence *”[Title/Abstract]))
Scopus	TITLE-ABS(“age friendly” OR “age friendliness” OR “elder friendly” OR “healthy aging” OR “aging-in-place” OR “urban aging” OR “active aging” OR “healthy ageing” OR “ageing-in-place” OR “urban ageing” OR “active ageing”) AND TITLE-ABS(intervention * OR initiative * OR policies OR policy OR framework *) AND TITLE-ABS(“older adult” OR senior * OR aged OR elder * OR geriatric *) AND TITLE-ABS(outcome * OR evaluation * OR impact * OR effect * OR benefit * OR evidence *)
ProQuest	ti(“age friendly” OR “age friendliness” OR “elder friendly” OR “healthy aging” OR “aging-in-place” OR “urban aging” OR “active aging” OR “healthy ageing” OR “ageing-in-place” OR “urban ageing” OR “active ageing”) AND ti(intervention? OR initiative? OR policies OR policy OR framework?) AND ti(“older adult” OR senior? OR aged OR elder?? OR geriatric) AND ti(outcome? OR evaluation? OR impact? OR effect??? OR benefit? OR evidence *) (ab(“age friendly” OR “age friendliness” OR “elder friendly” OR “healthy aging” OR “aging-in-place” OR “urban aging” OR “active aging” OR “healthy ageing” OR “ageing-in-place” OR “urban ageing” OR “active ageing”) AND ab(intervention? OR initiative? OR policies OR policy OR framework?) AND ab(“older adult” OR senior? OR aged OR elder?? OR geriatric) AND ab(outcome? OR evaluation? OR impact? OR effect??? OR benefit? OR evidence *))
Ebscohost AgeLine, CINAHL, CINAHL Complete, Medline, Medline Complete	(“age friendly” OR “age friendliness” OR “elder friendly” OR “healthy aging” OR “aging-in-place” OR “urban aging” OR “active aging” OR “healthy ageing” OR “ageing-in-place” OR “urban ageing” OR “active ageing”) AND (intervention * OR initiative * OR policies OR policy OR framework *) AND (“older adult” OR senior * OR aged OR elder * OR geriatric *) AND (outcome * OR evaluation * OR impact * OR effect * OR benefit * OR evidence *)
Embase	(‘age friendly’:ab,ti OR ‘age friendliness’:ab,ti OR ‘elder friendly’:ab,ti OR ‘healthy aging’:ab,ti OR ‘aging-in-place’:ab,ti OR ‘urban aging’:ab,ti OR ‘active aging’:ab,ti OR ‘healthy ageing’:ab,ti OR ‘ageing-in-place’:ab,ti OR ‘urban ageing’:ab,ti OR ‘active ageing’:ab,ti) AND (intervention *:ab,ti OR initiative *:ab,ti OR policies:ab,ti OR policy:ab,ti OR framework *:ab,ti) AND (‘older adult’:ab,ti OR senior *:ab,ti OR aged:ab,ti OR elder *:ab,ti OR geriatric *:ab,ti) AND (outcome *:ab,ti OR evaluation *:ab,ti OR impact *:ab,ti OR effect *:ab,ti OR benefit *:ab,ti OR evidence *:ab,ti)
Cochrane Library	(“age friendly” OR “age friendliness” OR “elder friendly” OR “healthy aging” OR “aging-in-place” OR “urban aging” OR “active aging” OR “healthy ageing” OR “ageing-in-place” OR “urban ageing” OR “active ageing”):ab AND (intervention * OR initiative * OR policies OR policy OR framework *):ab AND (“older adult” OR senior * OR aged OR elder * OR geriatric *):ab AND (outcome * OR evaluation * OR impact * OR effect * OR benefit * OR evidence *):ab (“age friendly” OR “age friendliness” OR “elder friendly” OR “healthy aging” OR “aging-in-place” OR “urban aging” OR “active aging” OR “healthy ageing” OR “ageing-in-place” OR “urban ageing” OR “active ageing”):ti AND (intervention * OR initiative * OR policies OR policy OR framework *):ti AND (“older adult” OR senior * OR aged OR elder * OR geriatric *):ti AND (outcome * OR evaluation * OR impact * OR effect * OR benefit * OR evidence *):ti

Note: The asterisk (*) is a search wildcard, representing any group of characters.

**Table A2:** Health and social outcomes of quantitative studies.

Author (Year)	Intervention	Outcome (1)	Outcome (2)	Outcome (3)	Outcome (4)	Outcome (5)	Outcome (6)
***Physical Activity Interventions***							
Ericson et al. (2018)	Resistance training program [P]	Increase in mean sense of coherence (SOC-13): 72.4 (pre), 72.9 (post)	Increase in mean health-related quality of life (SOC-13 (MCS): 52.0 (pre), 54.5 (post); (PCS): 53.9 (pre), 54.1 (post))	Increase in mean hope (HOPE): 43.9 (pre) 47.1 * (post)	Change in mean affect (PANAS (positive): 18.6 (pre), 18.9 (post); (negative): 10.3 (pre), 7.9 * (post))		
Liao et al. (2019)	Tai Chi program with background music [P]	Increase in mean physical domain (WHOQOL-BREF): 40.40 (pre), 52.89 ***(post)	Increase in mean psychological domain (WHOQOL-BREF): 44.72 (pre), 54.05 *** (post)	Increase in mean social domain (WHOQOL-BREF): 46.39 (pre), 57.40 *** (post)	Increase in mean environmental domain (WHOQOL-BREF): 45.02 (pre), 52.67 ***(post)		
Martins et al. (2020)	Exercise program [P]	Increase in functional abilities: hand strength *, 4-stage balance test ***, step test *, 30 s sit-to-stand *, timed up-and-go (n.s.)	Increase in self-efficiency for exercise (n.s.)	Improved participation performance (PAPM) ***			
McKay et al. (2018)	Physical activity program [P]	Increase in physical activity at 3 months (+1.6 days/week ***) and 6 months (+1.4 days/week ***) in younger group (60–74 years); increase in physical activity at 3 months (+1 days/week ***) in older group	Decrease in % reporting mobility limitation at 3 months (−15% ***) and 6 months (−12% ***) in younger group (60–74 years); decrease in % reporting mobility limitation at 3 months (−14% *) in older group (>75 years)	Decrease in sitting time at 3 months (−0.5 *) and 6 months (−0.4) in younger group; decrease in sitting time at 3 months (−0.2) and 6 months (−1.1 ***) in older group	Decrease in social exclusion at 3 months (+0.6 ***) and 6 months (+0.4 *) in younger group; no significant result in older group	Decrease in loneliness at 3 months (−0.4 ***) and 6 months (−0.3 ***) in younger group; decrease in loneliness at 3 months (−0.5 ***) and 6 months (−0.4 ***) in older group	Increase in happiness at 3 months (+0.2 ***) and 6 months (+0.1 ***) in younger group; increase in happiness at 3 months (+0.2 ***) and 6 months (+0.2 ***) in older group
McNamara et al. (2016)	Healthy aging activity program [P]	Increase in leisure activities (including health-related activities): 72% (pre), 77% (post)	Increase in social engagement: 70% (pre), 72% (post)				
Merchant et al. (2021)	Peer-led, dual-task exercise program [P]	Reduction in prevalence of depressive symptoms (Euroqol−5D domain): 30% **	Decrease in prevalence of fragility (FRAIL): 50–75% **	Decrease in prevalence of 1 or more falls: 66.66% (2/3) **	Decrease in social isolation (LSNS-6): 10% **		
Noh et al. (2020)	Exercise program [P]	Increase in mean physical health (skeletal muscle mass: 21.13 (pre), 21.47 *** (post); BMI: 23.32 (pre), 22.90 *** (post); body fat percentage: 32.09 (pre), 31.35 *** (post); waist circumference: 80.27 (pre), 78.63 *** (post); hip circumference: 95.05 (pre), 95.81 * (post); grip strength: 21.69 (pre), 23.35 *** (post); abdominal muscle strength: 14.62 (pre), 21.19 *** (post); flexibility: 14.09 (pre), 18.79 *** (post))	Decrease in prevalence of mean mental health symptoms (emotion (depression): 48.38 (pre), 43.57 * (post); emotion (anxiety): 51.71 (pre), 48.10 (post); emotion (phobic anxiety): 49.19 (pre), 46.10 * (post); emotion (panic attack): 51.43 (pre), 46.90 (post); emotion (agoraphobia): 47.38 (pre), 46.00 * (post); emotion (obsessive-compulsive): 49.76 (pre), 46.52 (post))	Decrease in prevalence of mean mental health symptoms (cont.) (emotion (obsession): 52.00 (pre), 48.29 (post); emotion (obsessive-compulsive personality trait): 46.90 (pre), 45.52 (post); emotion (PTSD): 49.05 (pre), 46.14 (post); emotion (aggression): 46.52 (pre), 44.86 (post); emotion (somatization): 51.00 (pre), 46.14 (post))	Decrease in prevalence of mental health symptoms (cont.) (adaptation to reality (manic episode): 51.71 (pre), 50.14 (post); adaptation to reality (paranoia): 46.38 (pre), 45.86 (post); adaptation to reality (schizophrenia): 48.81 (pre), 47.14 (post))	Decrease in prevalence of mental health symptoms (cont.) (others (suicide): 45.76 (pre), 44.67 (post); others (addiction): 46.76 (pre), 47.10 (post); others (sleep problem): 53.95 (pre), 50.57 * (post); others (stress vulnerability): 51.71 (pre), 48.10 * (post); others (self-regulation problem): 51.19 (pre), 45.86 * (post))	Decrease in negative social behaviors (emotion (social desirability): 54.00 (pre), 51.90 (post); emotion (inconsistency): 50.05 (pre), 51.90 (post); others (interpersonal sensitivity): 52.24 (pre), 49.00 (post))
Douka et al. (2019)	Greek traditional dance program [P]	Improvement in attention in both healthy and MCI groups (S4viac ***, S4viti ***)	Improvement in verbal fluency for MCI group (Verflx *, Verfls ***, Verfmo ***)	Improvement in executive functions (FUCAS *)	Improvement in anxiety for healthy * and MCI * groups	Increase in physical condition (arm curl ***, chair stand ***, back scratch+, 2 min step *, timed up-and-go ***, sit and reach ***, balance on one leg ***)	Increase in quality of life (healthy: 62.63 (pre), 64.88+ (post); MCI: 61.25 (pre), 65 * (post))
***Educational Interventions***							
Dumitrache et al. (2017)	Cognitive stimulation, crafts, and exercise workshops [E]	Increase in mean cognitive performance (MEC/MMSE: 27.29 *** (pre), 30.50 *** (post); CDT: 5.62 ***(pre), 7.17 ** (post))	Increase in mean health (WHOQOL-BREF (general): 3.38 (pre), 3.45 (post); (physical): 25.17 (pre), 25.30 (post); (psychological): 21.49 (pre), 21.91 ***(post))	Increase in mean quality of life (General QL): 3.28 (pre), 3.31 (post)	Social relationship mean remains constant (WHOQOL-BREF): 10.88 (pre), 10.88 (post)	Improved mean environment (WHOQOL-BREF): 25.10 (pre), 21.61 ***(post)	
Fernández-Ballesteros et al. (2012)	University programs [E]	Increase in mean cognitive functioning (digit-symbol): 44.36 (pre), 45.54 *** (post)	Decrease in mean illness reported (objective (illness reported): 0.79 (pre), 0.77 *** (post))	Increase in mean general health (subjective: 3.05 (pre), 3.06 (post); compared with others: 3.61 (pre), 3.64 (post))	Change in mean affect (positive affect, increase: 3.01 (pre), 3.15 *** (post); negative affect, decrease: 1.71 (pre), 1.65 ** (post); balance, increase: 1.84 (pre), 2.07 (post))	Increase in mean social relationships (social networks): 7.84 (pre), 7.85 (post)	Change in mean activity (information-seeking, increase: 3.34 (pre), 3.35 *** (post); social, increase: 2.29 (pre), 2.39 *** (post); productivity, decrease: 2.28 (pre), 2.24 *** (post))
Gough et al. (2017)	Peer-led education series [E]	Increase in awareness of brain health and resilience (FoH information): 50.0% (pre), 96.3% *** (post)	Increase in FoH information application 38.3% (pre), 92.6% (post)	Increase in awareness of interventions to promote cognitive and mental health: 32.1% (pre), 100% *** (post)	Increase in ability to name four or more evidence-based brain health and resilience interventions: 23.1% (pre), 90.0% *** (post)	Increased confidence in goal setting to promote personal health and well-being: 82.0% (pre), 92.3% * (post)	Improved attitude towards aging (average self-perception score): 3.08/5 (pre), 4.08/5 * (post)
Mendoza-Nunez et al. (2018)	Healthy aging education program [E]	Increase in health behavior (health care: 7.7 (pre), 8.2 * (post); self-esteem: 8.3 (pre), 8.4 (post); healthy food: 7.9 (pre), 8.1 (post); physical exercise: 8.0 (pre), 8.5 (post))	Increase in healthy lifestyle (sleep hygiene: 7.4 (pre), 8.2 * (post); body hygiene: 8.4 (pre), 8.9 (post); healthy environment: 8.4 (pre), 8.5 (post))	Increase in self-perception of aging (where having a positive self-perception (score ≤ 44) and negative self-perception (score ≥ 59): 51 (pre), 40 *** (post)			
Oetzel et al. (2020)	Peer education program [E]	Increase in self-rated health: +4.47 (average treatment effect on the treated, ATT)	Increase in health-related quality of life: +2.66 (ATT)	Increase in perceived support: 0.35 * (ATT)	Increase in desired support: 0.03 (ATT)	Increase in tribal identity: 0.36 * (ATT)	Decrease in loneliness: 0.05 (ATT)
Rabiei et al. (2013)	Family-based empowerment program [E]	Decrease in mean perceived threat (SF-36 (public health): 20.81 (pre), 14.33 ***(post); (physical problems): 21.25 (pre), 10.71 ***(post); (mental health): 35.55 (pre), 19.70 ***(post); (pain): 24.82 (pre), 13.57 ***(post); (general health): 21.04 (pre), 12.06 (post))	Decrease in mean perceived threat (SF-36 (playing role): 23.98 (pre), 14.95 ***(post); (social performance): 25.27 (pre), 15.50 ***(post); (power and energy): 18.91 (pre), 11.15 ***(post); (life quality): 17.30 (pre), 10.92 ***(post))				
Shinkai et al. (2016)	Health education program [E]	Increase in life-space mobility (men: 80.8% (pre), 86.9% (post); women: 63.7% (pre), 74.3% (post))	Increase in instrumental ADL (men: 72.9% (pre), 80.8% (post); women: 66.5% (pre), 76.3% (post))	Increase in mean intellectual activity (men: 64.2% (pre), 64.7% (post); women: 49.0% (pre), 57.2% (post))	Increase in social role (men: 55.0% (pre), 58.8% (post); women: 51.5% (pre), 58.3% (post))		
Tagliabue et al. (2018)	Multi-domain cognitive training [E]	Increase in global cognitive functioning (MoCA)	Non-significant changes in verbal and visuo-spatial memory (forward digit span *, backward digit span, forward Corsi span, backward Corsi span, short story test, recall of ROCF)	Non-significant changes in visuo-constructional abilities (copy of ROCF)	Increase in executive functions on most tests (semantic verbal fluency *, phonological verbal fluency, Stroop test (time) **, Stroop test (errors) *)	Increase in measures of attention (TMT); increase in non-verbal reasoning (Raven’s Matrices) +	Positive influence on mood: 57.53%; positive influence on socialization: 41.89%
***Multi-domain Interventions***							
Castle et al. (2008)	Senior living enhancement program (SLEP) [M]	Decrease in reported participation in regular activity (item 1a): 55% (pre), 68% * (post); increase in physical activity 1 or more times weekly (item 1b): 45% (pre), 55% * (post)	Increase in lack of knowledge pertaining to participants’ blood glucose level, LDL cholesterol level, and systolic blood pressure level (item 2a, 7, and 10a): 33% (pre), 52% *(post), 33% (pre), 53% (post), and 85% (pre), 86% * (post); increase in reported glucose level as ideal (<110) or moderate high (11–129), and systolic blood pressure as ideal (under 140) (item 2b and 10b): 14% (pre), 36% * (post), 51% (pre), 52% * (post)	Increase in engagement with preventative care (item 5a (sigmoidoscopy or colonoscopy in past 5 years): 42% (pre), 52% * (post); item 5b (skin examination in past year): 9% (pre), 25% (post); item 5c (fecal occult blood in past year): 30% (pre), 43% * (post); item 5d (PSA test in past year): 42% (pre), 58% * (post); item 5e (mammogram in past 1–2 years): 52% (pre), 66% (post); item 5f (PAP test in past 1–2 years): 38% (pre), 49% (post); item 6a (flu shot in past year): 55% (pre), 75% * (post); item 6b (pneumonia shot in past 10 years): 63% (pre), 74% * (post))	Improved smoking habits (item 3): 82% (pre), 84% * (post)	Decrease in the experience of muscle weakness or reduced muscle strength (item 9): 71% (pre), 61% * (post)	Increase in social activities outside the home (item 4a): 34% (pre), 60% (post); increase in strong social ties with friends/family: 70% (pre), 77% (post)
Park et al. (2011)	Health education and exercise program [M]	Decrease in mean blood pressure (systolic): 134.6 (pre), 122.3 **(post); (diastolic): 77.9 (pre), 77.0 (post)	Increase in mean physical activity (MET-min/week (vigorous intensity): 1180.0 (pre), 2248.9 (post); (moderate intensity): 475.5 (pre), 1275.3 (post); (walking): 1534.3 (pre), 1915.5 (post); (total physical activity): 2825.9 (pre), 4938.1 *(post))	Change in health-related quality of life (SF-36 (physical functioning): 80.3 (pre), 85.0 (post); (role-physical): 75.3 (pre), 79.5 (post); (bodily pain): 69.4 (pre), 66.1 (post); (general health): 51.7 (pre), 68.9 ** (post); (vitality): 57.9 (pre), 72.6 *(post); (mental health): 70.3 (pre), 81.7 * (post))	Increase in mean self-care behavior: 60.6 (pre), 67.6 * (post)	Increase in self-efficacy for exercise: 625.6 (pre), 643.3 (post)	Increase in mean health-related quality of life (SF-36 (social functioning): 65.0 (pre), 73.3 * (post); (role-emotional): 84.3 (pre), 87.9 (post))
Rainero et al. (2021)	Multi-center health promotion program [M]	Decrease in depressed mood *	Increase in positive nutritional habits **	No significant changes in physical and cognitive function, nor sleep.	No change in quality of life or social function		

Note: statistical significance, + *p* < 0.1, * *p* < 0.05, ** *p* < 0.01, *** *p* < 0.001; [P] denotes physical activity interventions; [E] denotes educational interventions; [M] denotes multi-domain interventions; and [O] denotes other interventions.

**Table A3:** Health and social outcomes of qualitative studies.

Author (Year)	Intervention	Health Outcomes	Social Outcomes
***Physical Activity Interventions***			
Haynes et al. (2021)	Physical activity and fall-prevention program [P]	The CHaNGE trial found an increase in reported physical activity, an increase in motivation and goal-setting behaviors, and an improvement for some participants in fitness and strength. Additionally, weight loss and increased fall prevention awareness or skills were also considered benefits of the program. The participants’ participation in the trial was measured using Fitbit technology, and many participants agreed that the accountability factor, in relation to tracking technology, increased their engagement in the intervention.	The trial found no change in social participation due to the intervention; however, “...one third of those who expressed a preference said they would like more interaction with their community group” (p. 299). Because the trial did not implement a social piece into their intervention, the participants relied on established connections as encouragement to engage in physical activity. The respondents who did engage in these established connections throughout the intervention reported a higher sense of security, companionship, mutual support, encouragement, and community.
VanRavenstein et al. (2018)	Physical activity program [P]	The participants within this program had differing responses dependent upon where they lived, as the intervention was provided in two separate senior living apartment complexes. The group which engaged in social behaviors throughout the intervention reported higher rates of engagement with the exercise and continuation of exercise post-program than the group that did not have regular socialization habits. The group which reported little socialization had a significant number of participants struggling with depression. This appeared to not be improved with the intervention.	A key aspect in this intervention was the participants’ relationship with socialization and their willingness to engage with other residents in the study. The group which actively participated in socialization practices had a different outlook on exercise and saw it as a fun activity and a way to encourage community.
***Educational Interventions***			
Morse et al. (2018)	Comedy improvisation course [E]	In the “Humor Doesn’t Retire” Program, respondents reported mood change, and more specifically a decrease in depressive symptoms. Other outcomes included in the study findings were “...enhanced mental flexibility and cognitive acuity following the program, accompanied by a willingness to accept the unexpected and react spontaneously” (p. 4).	The participants in this program cited many reasons as to why they engaged in this social intervention, with some attending to improve their social or communication skills as a way of self-development, while others attended due to their interest in the activities or desire to have fun. The study found that increased social participation/connection and decreased isolation were common themes amongst the respondent interviews.
Sims-Gould et al. (2020)	Health promotion program [E]	This study followed multiple Active Aging Grant (AAG) funded organizations and their implementation of health promotion programs within community settings. Participants from multiple organizations reported physical activity in the programs as “…beneficial, enjoyable, and at times, life altering” (p. 6). Despite each site having different physical activity programs, the older adults’ engagement and enjoyment of the program appeared to provide positive results.	Social activity and connectedness varied between the sites but were still reported to be positive by respondents. The social outcomes of this study included increased social connectedness for both the participants and the facilitators, as well as a decrease in social isolation and loneliness.
***Multi-domain Interventions***			
Greenfield et al. (2015)	Naturally occurring retirement community (NORC) support programs [M]	Through this study, the participants were able to access both preventative and supportive services to improve their overall health and well-being. Many participants in the study found these services to be vital in their ability to age-in-place in a healthy manner.	The NORC study revealed that older adults’ involvement in NORC programs is not just determined by their utilization level, but rather by their relationship with the NORC program as a whole. Some of the characteristics of high involvement included a close relationship with staff, lifelong histories, and a desire to ‘give back’ to the program.
Orellana et al. (2020)	Day centers for older adults [M]	Day centers for older adults were found to provide services which allowed participants to continue to age-in-place without requiring the individual to engage in a traditional residential care model. Due to the wide range of services and activities offered, many participants in this study reported an increase in mental well-being/health, physical well-being/health, as well as increased hygiene.	This study followed participants who were already established in day centers; therefore, social connectedness was based on the individual’s social engagement both in and out of the program. Social participation/involvement, feelings of dignity/control, quality of life, and feelings of security were all found to be common themes amongst those who attend day centers.

Note: [P] denotes physical activity interventions; [E] denotes educational interventions; [M] denotes multi-domain interventions; and [O] denotes other interventions.
